# Functional Characterization of the Hephaestin Variant D568H Provides Novel Mechanistic Insights on Iron-Dependent Asbestos-Induced Carcinogenesis

**DOI:** 10.3390/ijms26062607

**Published:** 2025-03-13

**Authors:** Paola Zacchi, Francesco Longo, Alice Marconato, Matteo Amadei, Maria Carmela Bonaccorsi di Patti, Elisa Avolio, Pengfei Li, Hongkuan Fan, Teresa D. Tetley, Giuliano Zabucchi, Violetta Borelli

**Affiliations:** 1 Department of Life Sciences, University of Trieste, 34127 Trieste, Italy; francesco.longo@studenti.units.it (F.L.); alice.marconato@studenti.units.it (A.M.); zabucchi@units.it (G.Z.); 2 Department of Biochemical Sciences, Sapienza University of Roma, 00185 Rome, Italy; matteo.amadei@uniroma1.it (M.A.); mariacarmela.bonaccorsi@uniroma1.it (M.C.B.d.P.); 3 Bristol Medical School, Translational Health Sciences, University of Bristol, Level 7 Bristol Royal Infirmary, Bristol BS2 8HW, UK; elisa.avolio@bristol.ac.uk; 4 Department of Pathology and Laboratory Medicine, Medical University of South Carolina, Charleston, SC 29425, USA; lippe@musc.edu (P.L.); fanhong@musc.edu (H.F.); 5 National Heart and Lung Institute, Imperial College London, Exhibition Road, London SW7 0HF, UK; t.tetley@imperial.ac.uk

**Keywords:** asbestos, iron, single nucleotide polymorphism, hephaestin, pericytes, mesothelioma, lung cancer

## Abstract

A local disruption of iron homeostasis leading to oxidative stress is considered one of the main mechanisms of asbestos-related genotoxicity. Another aspect contributing to the risk of developing pathological consequences upon asbestos exposure is individual genetic factors. In a previous study, we identified a coding SNP in the hephaestin gene (*HEPH*) that protects against developing asbestos-related thoracic cancer. Heph is a ferroxidase that promotes iron export in concert with the permease ferroportin (Fpn1). Here, we performed an in-depth functional characterization of the HephD568H variant to gain insights into the molecular basis of its protective activity. We showed that HephD568H forms a complex with Fpn1 and possesses full ferroxidase activity. Although HephD568H is more efficiently recruited to the plasma membrane, it is impaired in binding iron-deficient Tfn, whose interaction with wild-type (WT) ferroxidase emerged as a novel mechanism to perceive brain iron needs. Heph is expressed in the human lung by pericytes and fibroblasts, and lung pericytes were shown to respond to iron demand by upregulating the iron exporter pair. These results extend the paradigm of local iron regulation discovered at the blood–brain barrier to the pulmonary vasculature. Furthermore, they establish a mechanistic link between changes in iron sensing and the risk of developing asbestos-related malignancies.

## 1. Introduction

**Figure 1:**
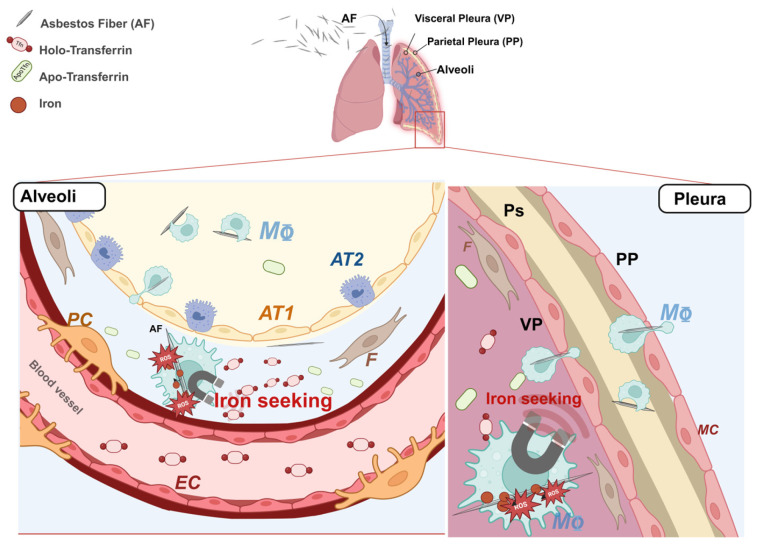
Schema summarizing the mechanism of asbestos-fiber-induced iron overload and consequent oxidative damage. Asbestos fibers reaching the alveoli and the pleural district are intercepted by alveolar macrophages. Once phagocytosed, asbestos fibers start to adsorb the host metal, leading the cell to develop an iron seeking phenotype. EC = Endothelial cell, F = Human lung fibroblast, PC = pericyte, AT1 = alveolar type 1 cell, AT2 = alveolar type 2 cell, MΦ = macrophage, MC = mesothelial cell, ROS = radical oxygen species, apo-Tfn = iron free transferrin, holo-Tfn = iron-bound transferrin, AF = asbestos fiber. Image created with Biorender.com.

The first medical article on the hazards of asbestos dust appeared in the *British Medical Journal* in 1924 and since then, several diseases related to asbestos exposure have been described [1]. Malignant pleural mesothelioma (MPM) and lung cancer (LC) are two types of neoplasms associated with occupational asbestos exposure [2,3,4] and due to the late and non-ubiquitous ban on asbestos and the long latency period of these diseases, they remain an urgent and current concern. Although a century has passed since the first report on the pathological consequences of asbestos exposure, the mechanisms underlying lung and pleural genotoxicity are still far from being understood. Currently, local disruption of iron homeostasis, which has been described both in animal models and in patients with asbestos-related cancers [5,6,7,8,9,10,11,12], and the resulting iron-induced oxidative stress are considered to be the main mechanisms of asbestos dependent genotoxicity [7,13,14,15,16,17]. Iron is a fundamental micronutrient all living cells utilize in various physiological processes. The ability of iron to gain and lose electrons, cycling between ferrous (Fe^2+^) and ferric (Fe^3+^) states, allows it to act as a co-factor for several iron-dependent enzymes involved in DNA replication and repair, cellular respiration and cell cycle progression [18]. However, an excess of redox-active iron can promote the generation of highly reactive oxygen species (ROS) which are potentially mutagenic due to their ability to damage DNA [19,20]. Iron is a structural component of amphibole minerals such as amosite, crocidolite and actinolite asbestos fibers. At the same time, serpentine chrysotile can induce hemolysis and thus iron release [21]. Once internalized by the cell, fiber-adsorbed iron acts as a catalyst in ROS production through the Fenton reaction [22,23]. In addition, internalized asbestos fibers can sequester the host metal on their surface, tricking the cell to perceive a functional iron deficiency [24,25] (Figure 1). In an attempt to restore iron homeostasis, the cell responds with either increased iron uptake and/or decreased iron egress, contributing to the establishment of a harmful iron-overload environment that triggers a cascade of cell signaling and the release of mediators leading to inflammation, fibrosis and neoplastic transformation [12,24,26] (Figure 1). Although mesothelioma has been considered for many years the paradigm of an environmentally induced cancer, diverse studies have suggested the presence of genetic predisposing factors in the etiology of the disease [27,28,29,30,31,32,33,34,35,36] and germline variants in the tumor suppressor gene BAP1 have been reported as a high-risk factor for MPM [37]. In the case of asbestos-associated LC, the contribution of genetics is still poorly understood. However, single genetic variations that may influence the risk of developing this neoplasm have been reported [38,39,40].

In a previous study, we used postmortem paraffin-embedded tissue to investigate the association between a whole set of iron metabolism genes and susceptibility to developing MPM and LC in a highly selected population exposed to asbestos [41,42]. Three SNPs, two non-coding in the ferritin heavy chain (FTH1) and transferrin (TF) genes and one coding SNP in the hephaestin gene (HEPH), were found to be protective against the development of both asbestos-related neoplasms. The coding SNP of the multicopper ferroxidase Heph has attracted our attention as it could be a potential prognostic indicator once a clear association between functional alterations of Heph, dysregulation of iron metabolism, and protection against MPM is demonstrated. Heph belongs to a small family of multicopper ferroxidases (MCFs) that promote the transfer of iron across biological membranes in concert with the multi-pass membrane protein ferroportin (Fpn1), the only known iron exporter in mammals [43,44]. The MCF family comprises three members, namely, ceruloplasmin (CP), hephaestin (Heph), and zyklopen (ZP) [45]. They are all characterized by multiple copper atoms that couple the oxidation of substrates with the complete reduction of oxygen to water [46]. By oxidizing ferrous iron to its ferric form, Heph improves the efficiency of iron export by Fpn1 and the iron loading of the major iron-carrier, the plasma glycoprotein transferrin (Tfn) [47]. Enterocytes exploit these activities to promote dietary iron absorption [48,49], but Heph expression is not restricted to the gastrointestinal tract. This ferroxidase has also been shown to be expressed by brain microvascular endothelial cells (BMVEC), where it controls iron delivery to the brain across the blood–brain barrier (BBB) [50,51]. In particular, iron demand is sensed by Heph interaction with the iron-depleted transferrin (apo-Tfn) [52], whose local levels raise when tissue iron consumption is elevated. Therefore, the Heph/apo-Tfn interaction would coordinate ferrous iron egress with its conversion into the Tfn loadable ferric form. In asbestos-related LC and MPM, the identified protective Heph SNP (rs3747359) introduces an aspartic acid replacement by histidine at position 568 (D568H) [41,42]. According to in silico analysis, this amino acid change is predicted to impact Heph function. We have recently addressed Heph distribution in the context of lung cancer and found that the protein is mainly expressed by vascular cells [53], a localization reminiscent of what has been observed in neuro-vascular units. In the vascular compartment of pulmonary tissue, Heph could exert a similar control over the local iron supply as in the brain, extending the paradigm uncovered at the BBB [52]. In this context, functional changes in the ferroxidase could affect iron procurement in response to a specific tissue demand and thus either favor or impede the development of deleterious iron overload.

In the present study, we performed an in-depth functional characterization of the HephD568H variant to gain valuable insights into the molecular basis of its protective activity in asbestos-related carcinogenesis. Our results showed that the amino acid change does not affect the ferroxidase activity of Heph or its ability to interact with Fpn1. Interestingly, although HephD568H/Fpn1 complexes were more enriched at the plasma membrane than HephWT, they were impaired in apo-transferrin recruitment and thus less prone to iron export. In the healthy human lung, endogenous Heph is expressed by two resident mesenchymal cell populations, namely, lung pericytes and fibroblasts: the former are functionally and physically associated with the vascular endothelium, while the latter are found directly beneath the alveolar epithelial cells or scattered in the interstitium. At this location, the expression of a Heph variant impaired in the ability to sense iron demand is likely to restrict iron supply in response to perceived local iron deficiency due to metal adsorption by fibers, thus preventing the development of an iron-induced oxidizing microenvironment, a fertile ground for neoplastic transformation. We propose that this mechanism may contribute to the observed protective effect of the identified non-synonymous substitution at position 568 of the Heph coding sequence against developing asbestos-induced cancers.

## 2. Results

### 2.1. HephD568H Distributes like HephWT When Ectopically Expressed in HEK293T Cells but Has a Prolonged Half-Life

**Figure 2:**
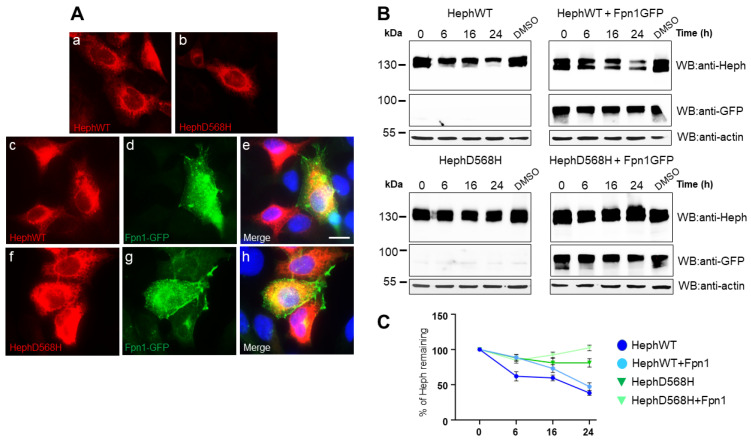
HephWT and D568H distribute similarly in HEK283T cells but are characterized by different half-lives. (**A**) Representative images of HEK293T cells transfected with HephWT and D568H either alone (panels (**a**,**b**)) or together with Fpn1-GFP (HephWT, panels (**c**–**e**); HephD568H, panels (**f**–**h**)). Heph distribution was analyzed using a monoclonal anti-Heph antibody followed by Alexa595-conjugated secondary antibody, while Fpn1-GFP was detected by the intrinsic green fluorescence of GFP. Scale bar = 10 µm; (**B**) HEK293T cells were transfected with 100 ng of HephWT or HephD568H either alone or in co-transfection with 100 ng Fpn1-GFP. In addition, 24 h post-transfection protein synthesis was blocked with cycloheximide (CHX) and the cells were harvested at the indicated time points. The amount of Heph protein remaining was analyzed by Western blot and densitometric scanning. Anti-actin was used as the loading control. (**C**) The results of three independent experiments are summarized in the graph, where the amount of HephWT or HephD568H proteins at time 0 was set as 100%. Standard deviations are indicated.

To gain insights into the molecular mechanisms underlying the “protective” anticancer activity of HephD568H, we initially investigated its intracellular distribution upon ectopic expression in HEK293T, a cell line lacking endogenous ferroxidase expression. Indirect immunofluorescence analysis performed 24 h after ectopic expression revealed no significant differences in subcellular distribution between HephD568H and the wild-type protein (Figure 2A, panels a and b). We then asked whether co-expression of Fpn1, the functional partner in the iron egress pathway, would affect the intracellular distribution and/or plasma membrane targeting of the ferroxidase variant, compared to HephWT, due to possible changes in Heph/Fpn1 binding affinity. Based on indirect immunofluorescence labeling, no significant differences in endomembrane distribution (Figure 2A, panels c–e HephWT; panels f–h HephD568H) and surface targeting were detected when the permease was co-expressed between WT and HephD568H, as judged by in vivo surface immunostaining (Figure S1). We then examined possible differences in half-life between the ferroxidases examined by Western blot analysis using the cycloheximide (CHX) chase assay. HEK293T cells were transfected either with individual Heph variants or together with Fpn1-GFP. Twenty-four hours after transfection, the cells were treated with 300 µM CHX and harvested at the indicated time points. Anti-Heph immunoblots showed that HephD568H protein levels remained elevated, independently of Fpn1-GFP expression, throughout all the time points examined. In contrast, HephWT halved after 16 h of treatment, but could be stabilized by the co-expression of Fpn1, which prolonged its half-life (Figure 2B,C).

### 2.2. HephD568H Interacts with the Permease Fpn1 but the Complex Is More Enriched at the Cell Surface with Respect to HephWT

Co-immunoprecipitation studies were then performed to test whether the amino acid substitutions to Heph would affect the interaction between Heph and Fpn1. Specifically, HEK293T cells were co-transfected with Fpn1-GFP and HephWT or HephD568H. Forty-eight hours after transfection, the GFP-trap, an anti-GFP nanobody with high binding affinity coupled to agarose beads, was used to precipitate over-expressed Fpn1-GFP from cell lysates selectively. In addition, a chemical cross-linking approach was used to reliably determine the amount of Heph/Fpn1 complexes formed before detergent-mediated lysis of the transfected cells. Two types of crosslinker were used: Lomant’s Reagent (DSP; dithiobis[succinimidyl propionate]), a cleavable crosslinker that is permeable to the cell membrane, and DTSSP (3,3’-Dithiobis[sulfosuccinimidylpropionate]), which, in contrast, is membrane-impermeable. These reagents were selected with the aim of not only evaluating possible differences in the ability of the ferroxidases to interact with Fpn1 but also to determine how these complexes are distributed between the plasma membrane, where the permease in conjunction with a ferroxidase exerts its role as iron exporter, and intracellular membrane compartments. Precipitated Fpn1-GFP was visualized by Western blotting with anti-GFP immunodetection, while co-interacting Heph variants were revealed by the anti-Heph monoclonal antibody.

**Figure 3:**
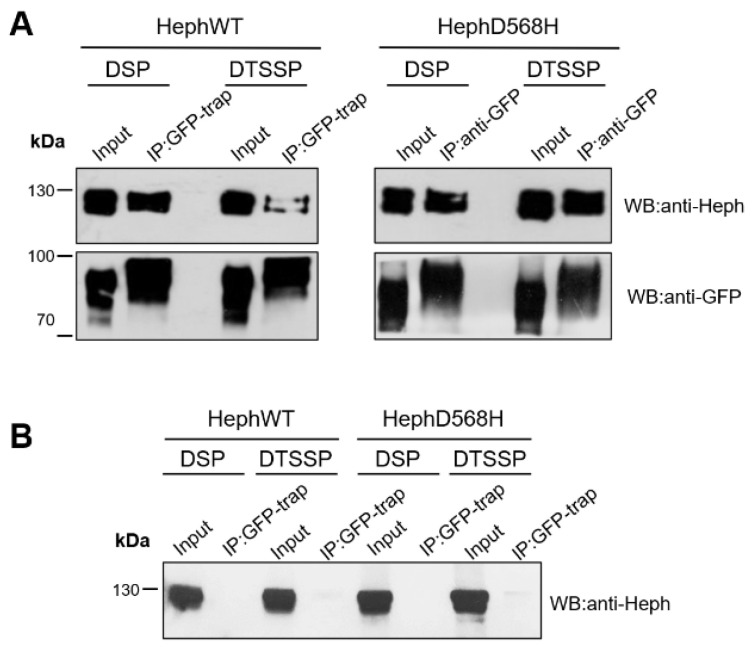
HephD568H/Fpn1 complexes are enriched at the plasma membrane. (**A**) HEK293T cells were co-transfected with either HephWT or HephD568H together with Fpn1-GFP. In addition, 24 h after transfection, cells were cross-linked as indicated. Cell lysates were incubated with GFP-trap agarose beads. Immunoprecipitate (IP) and 10% of cell lysate (input) were processed for immunoblotting. Immunoprecipitation of Fpn1-GFP shows that both HephWT and HephD568H are pulled down along with the Fpn1 complex upon DSP treatment, while DTSSP surface cross-linking indicates that HephD568H/Fpn1 complexes are enriched at the surface as compared to HephWT. Western blots were performed with anti-GFP monoclonal and anti-Heph monoclonal antibodies (n = 4). (**B**) HEK293T cells were only transfected with HephWT or HephD568H alone and processed as described in (**A**). In the absence of Fpn1-GFP co-transfection, over-expressed HephWT and HephD568H are not specifically pulled-down by GFP-Trap agarose (n = 4).

As shown in Figure 3A, HephD568H was able to interact specifically with Fpn1-GFP just like the WT ferroxidase, as no Heph pull-down was observed in the absence of Fpn1-GFP co-transfection (Figure 3B). Interestingly, the band intensity of Heph co-precipitated in DSP- and DTSSP-treated cells was comparable for HephD568H, indicating that these complexes are largely surface exposed. In contrast, the band intensity of HephWT appeared much higher in DSP-treated cells compared to DTSSP-treated cells (Figure 3A, lanes 2 vs. lane 4). Overall, these results suggest that although the amino acid substitution does not affect the ability of Heph to form a complex with the permease, the Heph variant/Fpn1 complexes are more efficiently transported to and/or retained at the cell surface than the wild-type ferroxidase. In this context, the observed differences between the half-life of HephWT and HephD568H could also play a functional role in the plasma membrane localization of the complex.

### 2.3. HephD568H Is Not Impaired in the Ferroxidase Activity

**Figure 4:**
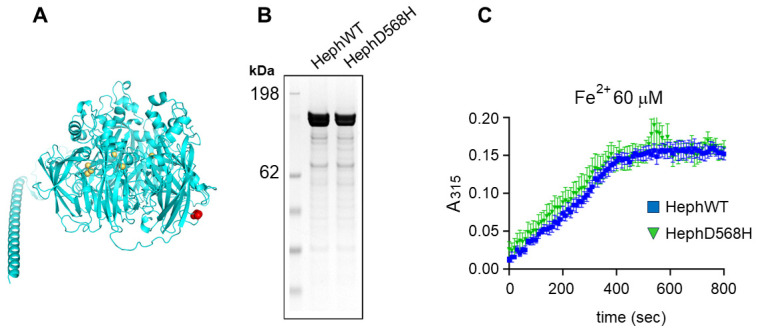
The ferroxidase activity is not affected in HephD568H. (**A**) Alphafold structural model of human Heph; residue D568 is colored in red and the Cu atoms are in orange. (**B**) SDS-PAGE analysis of purified human Heph WT and HephD568H. The gel was stained with Coomassie Blue. (**C**) Time-course of ferroxidase activity measured at 315 nm with 60 µM Fe(NH_4_)_2_(SO_4_)_2_ and 0.3 µM recombinant protein.

We then asked whether the amino acid substitution could affect the ferroxidase activity of the mutant. The position of residue D568 is predicted to be at the protein’s surface, in a loop in the basal region of Heph (Figure 4A). The target amino acid is not involved in copper coordination/binding and is located far from the predicted iron-binding sites, suggesting that it has little, if any, effect on ferroxidase activity. Recombinant full-length HephWT and HephD568H were produced in Expi293F cells and purified as described in the Materials and Methods (Figure 4B). Both WT and mutant Heph were enzymatically active, as measured at 315 nm with 60 µM ferrous iron (Figure 4C). Time course measurements can be used to extract the kinetic parameters Km and Vmax based on an integrated form of the Michaelis–Menten equation [54]. The time course of iron oxidation by WT and mutant Heph was analyzed using iFIT [55] and the kinetic parameters are listed in Table 1. Km and Vmax for HephWT are consistent with the values at a low iron concentration reported for the recombinant protein expressed in BHK cells [56]. The results indicate that the mutation appears to have no impact on Vmax, while the Km is slightly, but not significantly, decreased compared to WT (Table 1).

### 2.4. HephD568H Affects Iron Sensing

**Figure 5:**
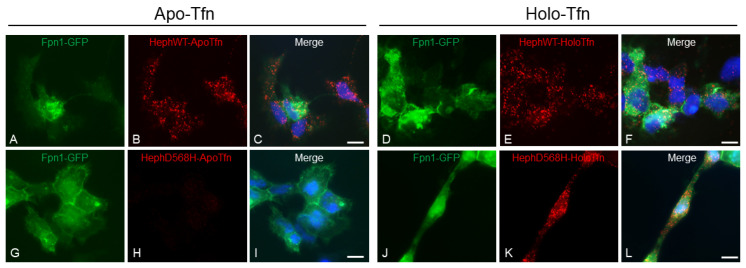
HephD568H variant is hampered in apo-Tfn recruitment. Duolink proximity ligation assay (PLA) was exploited for detecting apo-Tfn and holo-Tfn interaction with HephWT (panels (**A**–**C**) and (**G**–**I**), respectively) and HephD568H (panels (**D**–**F**) and (**J**–**L**), respectively). HEK293T cells co-transfected with Fpn1-GFP and HephWT or HephD568H were treated with 0.25 μM apo-Tfn or holo-Tfn for 30 min prior to fixation. PLA detects HephWT’s ability to interact with apo-Tfn, while HephD568H is strongly impaired. PLA signal detected upon holo-Tfn incubation is attributed to Fpn1 vicinity to Heph. Nuclei were stained with 4, 6-diamidino-2-phenyl indole (DAPI) in blue. Scale bar: 10 µm. Representative images are shown from three independent experiments. Positive and negative controls used for assay optimization can be found in Figure S2.

Recently, it has been shown that the release of iron into the brain extracellular fluid is finely regulated by Tfn, the principal iron cargo molecule, via its differential binding to Heph and Fpn1 [52]. In particular, it has been observed that when brain iron demand is high, apo-Tfn concentration in the microenvironment increases. Heph, which is expressed by brain microvascular endothelial cells, can sense these changes and recruit apo-Tfn: this allows for the optimization of ferrous iron egress via Fpn1 and its subsequent oxidation and loading onto Tfn. Conversely, the increased concentration of iron-loaded Tfn (holo-Tfn) in an iron-rich environment impedes the further release of iron upon binding of Fpn1, leading to its internalization, ubiquitination and proteasomal degradation [51]. We therefore sought to investigate the ability of HephD568H, which is enriched at the plasma membrane in transfected cells compared to HephWT, to recruit apo- and holo-Tfn using a highly sensitive assay for detecting protein–protein interactions, the proximity ligation assay (PLA). For this purpose, HEK293T cells were co-transfected with Fpn1-GFP and either HephWT or HephD568H: twenty-four hours after transfection, the cells were incubated for 30 min with 0.25 μM apo-Tfn or holo-Tfn dissolved in serum-free medium. Only cells transfected with HephD568H showed very few PLA puncta when assayed for apo-Tfn and Heph interactions (Figure 5, panel H) compared to HephWT, which showed a strong PLA signal (Figure 5, panel B); PLA puncta were instead detected in HephWT or HephD568H transfected cells incubated with holo-Tfn and assayed for holo-Tfn-Heph interactions (Figure 5, panel E and K). We interpreted this result not as evidence for the ability of Heph to recruit iron-loaded Tfn, regardless of the variant tested, but as a consequence of the proximity between Heph and holo-Tfn interacting with Fpn1, which is known to form a complex with ferroxidase at the cell surface.

**Figure 6:**
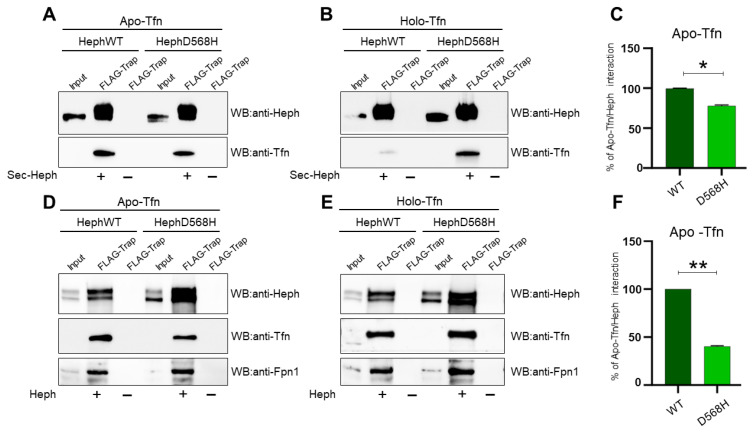
Apo-Tfn is poorly recruited by HephD568H as compared to HephWT. (**A**,**B**) Representative IP of FLAG epitopes from medium of HEK293T cells expressing the secreted extracellular domain of HephWT and HephD568H. Each affinity resin has been split to be further incubated with apo-Tfn and holo-Tfn. Nitrocellulose membranes were probed with anti-Heph and anti-Tfn antibodies. (**C**) The histogram on the right shows the relative amount of apo-Tfn co-precipitated by HephWT and HephD568H (n = 4, data are expressed as mean ± SEM; * *p* < 0.05). (**D**,**E**) Representative co-IP of FLAG epitopes of HEK293T cells transfected with HephWT and D568H and undergone DSP cross-liking before lysis. The immunoprecipitated Heph/Fpn1 complexes were split to be further incubated with apo-Tfn or holo-Tfn. (**F**) The histogram on the right shows the relative amount of apo-Tfn co-precipitated by HephWT and HephD568H (n = 5, data are expressed as mean ± SEM; ** *p* < 0.01). The amount of apo-Tfn co-precipitated by HephWT and HephD568H, either secreted or inserted into the plasma membrane, was quantified by densitometric scanning of Western blot. The amount of apo-Tfn interacting with HephWT was set as 100% interaction.

To further investigate the binding specificity revealed by the PLA assay, we decided to set up an experimental system that would provide the capacity to reliably test equal amounts of Heph variants against apo- and holo-Tfn, a condition not easily achieved in cell transfection approaches. With this aim, the FLAG-tagged extracellular domains of HephWT and HephD568H were over-expressed in HEK293T cells and the corresponding secreted extracellular domain (sec-Heph) was purified from the culture medium using the Chromoteck FLAG-trap. Equal amounts of each affinity resin were then used to pull down either apo- or holo-Tfn and the pulled-down fractions were quantified using Western blot analysis. As shown in Figure 6, sec-HephWT confirmed the ability to recruit apo-Tfn, while sec-HephD568H showed a small, albeit significant, decrease in apo-Tfn interaction (Figure 6A,C). Unexpectedly, we also observed recruitment of holo-Tfn by sec-HephD568H, while sec-HephWT, as expected, confirmed its selectivity only for iron-depleted Tfn (Figure 6B).

These results alerted us that the isolated extracellular domain of ferroxidase, which is less constrained than the membrane-tethered form, might provide non-physiologically relevant epitopes for apo- and holo-Tfn capture. We, therefore, decided to test Tfn binding in co-immunoprecipitation experiments after the expression of full-length Heph in HEK293T. Transfected cells were therefore incubated for 30 min with 0.25 μM apo-Tfn or holo-Tfn dissolved in serum-free medium, as was the case for the PLA assay. After Tfn binding, a chemical crosslinking approach was also employed before lysing the cells to preserve the integrity of the Heph/Fpn1/Tfn tripartite complex before DYKDDDDK Fab-Trap immunoprecipitation. Regardless of whether the cells were transfected with a specific Heph variant, holo-Tfn was similarly co-precipitated with anti-FLAG beads due to its interaction with Fpn1 (Figure 6E). In contrast, upon binding of apo-Tfn, we observed poor recruitment by HephD568H compared to HephWT, as shown in the PLA (Figure 6D,F).

Overall, these findings suggest that HephD568H, which is characterized by a limited ability to interact with iron-depleted Tfn, may consequently impact Tfn iron loading, thus affecting the local iron supply.

### 2.5. Heph Is Expressed by Human Lung Pericytes and Fibroblasts

To understand the contribution of HephD568H to developing asbestos-related cancers, it is necessary to identify the cell types generally involved in ferroxidase expression. We have recently shown that Heph is mainly expressed by the pulmonary vasculature of peritumoral tissues in the context of lung cancer [53], a distribution reminiscent of what has been observed at the neurovascular unit, where Heph contributes to the regulation of iron supply to the central nervous system [52,57]. To test whether this vascular distribution is also present in the normal lung, we performed Western blot analysis after surface biotinylation of primary human lung endothelial cells and immortalized human lung pericytes [58], mural cells particularly abundant in the lung, correlating with a stronger barrier and lower turnover of endothelial cells [59]. We also included primary human lung fibroblasts in the analysis, which were identified as Heph-expressing cells in our initial immunohistochemical localization studies performed on lung adenocarcinoma and squamous cell carcinoma based solely on their typical elongated spindle-shaped morphology [53]. To complete the overall view of Heph expression in the pulmonary and pleural districts, the sites of major asbestos-related neoplasia, the expression of Heph/Fpn1 iron egress complex was also examined in TT1, HMC and MeT-5A cells (Figure S3 panel A).

**Figure 7:**
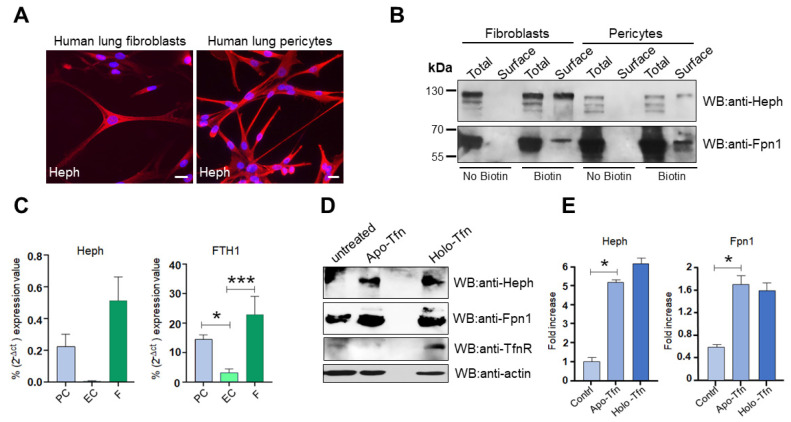
Heph is expressed by lung fibroblasts and pericytes and is up-regulated in response to apo-Tfn. (**A**) Representative epifluorescence images of primary lung fibroblasts and pericytes stained for Heph expression. Nuclei were stained with 4, 6-diamidino-2-phenyl indole (DAPI) in blue. Scale bar = 10 µm. (**B**) Heph and Fpn1 derived from cultured lung fibroblasts and pericytes were detected upon surface biotinylation assay and detected by anti-Heph and anti-Fpn1 monoclonal antibodies. Non-biotinylated cells were processed in parallel to evaluate unspecific Heph and Fpn1 binding to neutravidin beads (n = 4). (**C**) Heph and ferritin heavy chain (FTH1) expression in lung pericytes (PC), lung endothelial cells (EC) and lung fibroblasts (F) were analyzed by qRT-PCR. Glyceraldehydes-3-phosphate dehydrogenase (GAPDH) was used as internal reference gene (n = 4, data are expressed as mean ± SEM; * *p* < 0.05, *** *p* < 0.001). (**D**) Cultured lung pericytes were treated with 0.25 µM apo-Tfn or holo-Tfn of left untreated. Cells were harvested 72 h after treatment and equal amount of total protein was run on an 8% SDS gel. Protein lysates were analyzed by Western blot for Heph, Fpn1 and TfnR expression. (**E**) The histograms on the right show the up-regulation of Heph and Fpn1 expression observed upon apo-Tfn and holo-Tfn treatment. Western blot bands were quantified by densitometric scanning and expressed as fold increase respect to untreated cells (n = 3, data are expressed as mean ± SEM; * *p* < 0.05).

Initial immunofluorescence analyses and qRT-PCR attested that human lung pericytes and human lung fibroblasts express the ferroxidase Heph (Figure 7A,C, left panel). Surface biotinylation, followed by Western blot analysis, confirmed the expression of Heph and targeting to the cell surface, in association with the permease Fpn1, by these mesenchymal cell types (Figure 7B). Evidence that pericytes are a Heph-expressing cell type is further supported by transcriptomic studies of primary pericytes isolated from human hearts (NCBI GEO Accession numbers GSE195917 and GSE218644). Although they lacked Heph expression, endothelial cells and all other cell lines tested were able to express the permease Fpn1, suggesting that they can perform iron export relying on ferroxidase activity provided by either neighboring cells or soluble CP, as Western blot analysis showed that they also lacked membrane-tethered CP expression. In addition, we observed that lung pericytes and fibroblasts express ferritin heavy chain significantly more than lung endothelial cells, suggesting an essential contribution to local iron storage (Figure 7C, right panel).

Finally, we wanted to investigate whether these cells, provided of the iron export route, would sense iron request and would be able to respond to it. A plausible response to increased iron demand would likely involve up-regulation of ferroxidase, the actual driver of iron export. Therefore, pericytes were treated for 72 h with apo-Tfn, to mimic a state of iron demand, or with holo-Tfn, to induce cellular iron overload. Cells were then harvested and tested for Heph expression. Western blot analysis unveiled that Heph expression was upregulated upon apo-Tfn incubation compared to untreated cells (Figure 7D,E). Ferroxidase levels also increased with holo-Tfn treatment but in this case, such enhanced expression was accompanied by a concomitant up-regulation of the Tfn receptor (TfnR), attesting an iron uptake exceeding the iron storage capacity of the cell, which could be then forced to export the excess. Up-regulation of TfnR did not occur in the case of apo-Tfn incubation, proving that the increased Heph expression was induced to satisfy a specific iron request.

## 3. Discussion

In the present study, we provide a new mechanistic understanding of how the Heph variant D568H, identified in a genetic susceptibility study as protective against the development of asbestos-dependent neoplasia, exerts its activity. This Heph variant can complex with Fpn1 and has full ferroxidase activity, similar to the WT form. In contrast, HephD568H has a longer half-life than HephWT and is more enriched at the plasma membrane coupled to the permease. Despite its enhanced surface recruitment, HephD568H is hampered in apo-Tfn binding, whose interaction with WT ferroxidase represents a newly identified regulatory mechanism of iron release at the BBB [52]. In the human lung, Heph is expressed by two resident mesenchymal cell populations, namely, lung pericytes and fibroblasts. Here, the presence of a Heph variant impaired in sensing local iron demand is expected to prevent the establishment of a harmful iron-overload condition promoted by respiratory cells, which perceive an iron deficiency following the adsorption of host metal to asbestos fibers. These data support the notion that changes in iron sensing are critical to an individual’s risk of developing asbestos-dependent neoplasms.

Heph was discovered to be the mutant gene responsible for the sex-linked anemia (*sla*) phenotype in mice [48]. The intestinal enterocytes of *sla* mice are impaired in iron delivery into the blood, leading to iron accumulation in the duodenum and thus to anemia. At the BBB level, Heph expressed by microvascular endothelial cells, astrocytes and pericytes supports brain iron supply precisely satisfying local demands [57,60]. This controlled release is achieved by interaction of ferroxidase with apo-Tfn, whose levels increase when holo-Tfn is utilized. This mechanism has been characterized in induced pluripotent stem cell (iPSC)-derived endothelial cells [61], a widely recognized in vitro model of the BBB [62,63,64]. In contrast, the contribution of pericytes to iron handling in the neuro-vascular unit has been poorly investigated, although these cells provide structural and nutritional support to endothelial cells contributing to the barrier function of the BBB [65,66]. These mural cells have been shown to co-express Heph with CP, both in soluble and GPI-anchored forms, just like astrocytes, but how their activity is coordinated with that of endothelial cells is largely unknown [57].

Our initial study addressing the possible contribution of Heph to lung tumor progression [53] showed that its expression is associated with the pulmonary vasculature and interstitial fibroblasts. This initial characterization of lung cancer specimens did not allow for a thorough characterization of the cell types expressing Heph and, more importantly, for researchers to address its distribution in a healthy context. To address this knowledge gap, we investigated Heph expression in primary human cell lines representative of healthy lung and pleural districts. Specifically, we tested alveolar type 1-like TT1 cells and microvascular endothelial cells, both integral parts of the alveoli–capillary barrier [67]; we included lung pericytes, which are fundamental to pulmonary vasculature physiology [68], lung fibroblasts, which provide extracellular matrix but also paracrine cues to the developing epithelium and endothelium, and, finally, primary human pleural mesothelial cells.

**Figure 8:**
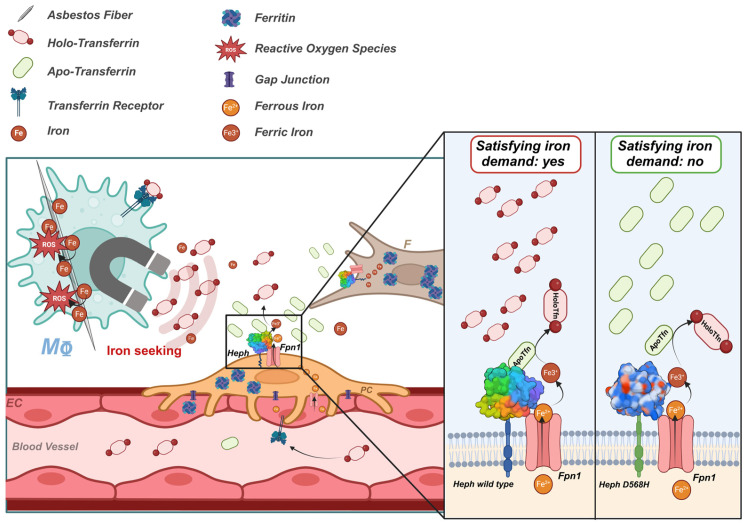
Model of how HephWT and D568H respond to iron demand. Asbestos fibers endocytosed by macrophages (MΦ) actively subtract host iron, thus provoking a homeostatic response aimed at re-establishing the appropriate cellular iron concentration. This iron-seeking phenotype depletes holo-Tfn and increases apo-Tfn levels. This iron demand is expected to be intercepted by nearby pericytes and fibroblasts, which are equipped to appropriately sense it. HephD568H impaired in the apo-Tfn interaction will respond inefficiently to the increased iron demand, thus hindering the development of a dangerous iron overload condition. EC = endothelial cell, F = human lung fibroblast, PC = pericyte, MΦ = macrophage, ROS = radical oxygen species, Heph = Hephaestin and Fpn1 = Ferroportin. Image created with Biorender.com.

Surface biotinylation experiments revealed that Heph at the pulmonary barrier is exclusively expressed by lung pericytes and fibroblasts. In contrast, pulmonary microvascular endothelial cells were negative for Heph (Figure S3B, left panel) and CP expression. This was further confirmed by qRT-PCR (Figure S3B, right panel). We also tested endothelial cells from human umbilical veins (Huvec), a physiologically relevant model system for vascular biology research. These cells lack Heph (Figure S3C, left panel) and rely on CP as ferroxidase for iron excretion (Figure S3C, right panel). These observations suggest that iron handling in the vascular units of different organs must be tailored to context-specific needs. The brain is one of the organs with the highest energy requirements and its iron needs not only support basic cellular processes but are also essential for neuron specific functions [69,70]. On the other hand, the lungs are extremely susceptible to metal-induced oxidative stress due to their high oxygen partial pressure [71]. In addition, excessive iron bioavailability could favor lung infections [72]. Therefore, as a protective strategy to prevent redox damage and potential microbes’ proliferation, lung epithelial cells maintain an intracellular iron concentration sufficient to meet their metabolic needs [73]. In this context lung pericytes, which have been shown to express Heph/Fpn1 complexes, rather than endothelial cells as found at the BBB, may be the primary cell type controlling iron mobilization upon local request. These mural cells also express more ferritin than lung endothelial cells, as determined by qRT-PCR, indicating a better iron storage capacity. It should be emphasized that an increase in iron demand in the pulmonary context is a rather exceptional event that serves to avoid any possible risk of iron-dependent oxidative damage. Inhaled asbestos fibers reaching the alveoli represent an unusual event that dramatically disrupts this delicate system in exposed cells, primarily in professional phagocytes such as alveolar macrophages. The sequestration of host metal by the fiber surface causes the cell to perceive a functional iron deficiency. This event triggers a homeostatic response to restore the appropriate cellular iron concentration. It can be assumed that such an increased local iron demand causes an enhanced utilization of holo-Tfn and a concomitant increase in apo-Tfn concentration (Figure 8). This iron demand is expected to be intercepted by nearby pericytes, which can sense it and respond by enhancing Heph and Fpn1 expression (Figure 7D). In case of a persistent iron demand, as occurs upon deposition of bio-persistent asbestos fibers, the prompt response to this demand likely results in local iron overload. However, if the mechanism of the iron supply does not respond, such a dangerous condition is more challenging to achieve. Since the HephD568H variant is protective against asbestos-dependent carcinogenesis, we hypothesized that its action might be due to the limitation of iron release in response to a sustained demand. This could be achieved by several mechanisms: impairment of Heph’s catalytic activity, alteration of Heph’s ability to associate with Fpn1 or defective capacity as an iron sensor.

In vitro characterization of ferroxidase activity demonstrated that the kinetic parameters did not differ significantly between HephWT and D568H. This could be because Asp 568 is located in a surface-exposed loop, away from the Cu-binding site. Replacing Asp with His removes a negative charge and this substitution is more likely to affect the stability of Heph and/or the ability to interact with molecular partners. Co-immunoprecipitation experiments showed that HephD568H/Fpn1 complexes were more enriched at the plasma membrane compared to the HephWT/Fpn1. Although the mechanisms involved in controlling intracellular association with Fpn1 and its targeting to the cell surface are still poorly understood, our results suggest that the stability of Heph is an important factor. HephWT, which is characterized by a shorter half-life compared to HephD568H, is poorly delivered to the plasma membrane. HephD568H, which is intrinsically more stable than the WT form regardless of Fpn1 expression, is more efficiently delivered to the cell surface.

We then tested the ability of apo- and holo-Tfn to bind Heph in immunoprecipitation experiments. We found that apo-Tfn was poorly recruited by HephD568H compared to the WT form, which was also confirmed by PLA. This phenotype was more evident for HephD568H expressed in a cellular context than for the corresponding secreted extracellular domain which also appeared to recruit iron-loaded Tfn in the absence of Fpn1. We hypothesized that the secreted HephD568H domain, which is endowed with a greater degree of freedom, would interact with holo-Tfn via protein interaction domains that are inaccessible when the ferroxidase is membrane-bound. Alternatively, these data could suggest that HephD568H does not efficiently release Tfn upon iron oxidation and loading. In this way, ferroxidase could act as an inefficient sensor because it is impaired in capturing apo-Tfn and keeps holo-Tfn sequestered. This behavior was further underscored with the secreted form of Heph, in the absence of Fpn1, the physiologically relevant binder of holo-Tfn. The molecular dissection of all steps involved in iron transfer from the Heph/Fpn1 complex to Tfn cannot preclude an in-depth structural characterization of the domains involved. Based on our results, we can confidently confirm that HephD568H is hampered in apo-Tfn recruitment, a condition that renders the ferroxidase poorly responsive to iron demand (Figure 8).

Regarding the contribution of lung fibroblasts to the phenotype, the fact that they express Heph and Fpn1 means that they are potentially able to respond similarly to lung pericytes, from which they may also be derived [74]. This ability has been described in gastric-cancer-associated fibroblasts, in which up-regulation of Heph and Fpn1 induces iron-overload-dependent ferroptosis in NK cells, thus hampering their anti-tumor immune response [75]. Based on qRT-PCR, fibroblasts can be considered an iron-storing cell type as they express more ferritin than pericytes and lung endothelial cells. Pericytes and resident fibroblasts are engaged in sensing and responding to changes in lung health and function through different mechanisms [76]. A better understanding of the relative contribution of pericytes and fibroblasts to regulate iron release in response to local demands still deserves further studies.

Another puzzling aspect is how lung pericytes and fibroblasts acquire iron since they are not in direct contact with the bloodstream. At least three possibilities are conceivable. In the first scenario, the iron taken up by the endothelium is exported by Fpn1 on the abluminal side of the endothelial membrane [50]. According to our findings, lung endothelial cells lack ferroxidase activity, which could be supplied by nearby pericytes/fibroblasts. Another possible route of iron supply could be the delivery of iron bound to Tfn and ferritin packaged in exosomes, a process recently described in the context of the BBB [77]. Interesting, ferroxidase activity was associated with exosomes isolated from mice exposed to asbestos fibers and identified as part of a unique protein signature [78]. Thirdly, iron could directly shuttle from the endothelial cell to the pericyte via gap junctions that directly connect the cytoplasm of both cell types [79].

Regardless of the mechanism(s) involved, the novelty of our study relies upon having attributed to lung pericytes and fibroblasts a key role in sensing local iron request. We also extended the paradigm discovered at the BBB to the pulmonary barrier, highlighting organ-specific features. Within this framework, the protective activity of the identified Heph genetic polymorphism could be reliably interpreted. The main limitation of our study is the lack of data on the localization of Heph in healthy pulmonary human subjects, attesting its expression in mesenchymal cells, an aspect that requires further investigation. We are confident that our results have improved our understanding of the genetic contributions to asbestos-dependent cancers and highlighted the importance of resident mesenchymal populations as targets for developing new prevention approaches in asbestos-exposed individuals.

## 4. Materials and Methods

### 4.1. Plasmids and Mutagenesis

The pCMV6 plasmid containing the entire sequence of the human HEPH ORF (NM_138737) with a C-terminal MYC-DDK tag [myc-DDK-HEPH1, hereafter WT (wild type)-HEPH] was obtained from Origene (RC215550, Origene Technologies, Rockville, MD, USA). Full-length HephWT with a C-terminal FLAG-tag and the point-mutant D568H, were generated by polymerase chain reaction (PCR) and cloned into pcDNA3. A truncated soluble version of Heph comprising the extracellular enzymatically active domain (residues 1-1107) and lacking the transmembrane and cytoplasmic domains was also produced and cloned into pCMV-Tag4b vector. For recombinant expression and purification, human HephWT and D568H full-length coding sequences were cloned in pOPINEneo-3C-TGP-His vector (MPL) by ligation-independent cloning (ClonExpress^®^ IIOne-Step Cloning kit, Vazyme, Oxford, UK). All PCR-amplified products were fully sequenced to exclude the possibility of second site mutations. The plasmid for expression of human Fpn1 tagged with GFP is described in the work of Bonaccorsi di Patti and colleagues [80].

### 4.2. Chemicals and Antibodies

Apo-Tfn, holo-Tfn and cycloheximide (CHX) were purchased from Sigma (#T1147, #T4132, #C1988, respectively, St. Louis, MO, USA).

The following primary antibodies were used in Western blot analysis: mouse monoclonal anti-Heph (1:1000; #sc-365365, Santa Cruz Biotechnology, Dallas, TX, USA), monoclonal anti-Fpn1 clone 31A5 kindly provided by Amgen (Thousand Oaks, CA, USA, 1:5000), polyclonal anti-Transferrin (Tfn) antibody (1:1000; #17435-1-AP, Proteintech, Rosemont, IL, USA), monoclonal anti-GFP antibody (1:1000, #MA5-15256, Invitrogen, Carlsbad, CA, USA) and anti-beta actin HRP-conjugated monoclonal antibody (1:5000; #sc-47778 HRP, Santa Cruz Biotechnology).

### 4.3. Cell Culture and Transfections

TT1 cells, an immortal alveolar type 1(AT1)-like cell line [67], were cultured in Hybridoma Serum Free Medium (#12045-084, Gibco, Grand Island, NY, USA) supplemented with 10% new-born calf serum (NCS, #N4762-500ML, Sigma) and 1% penicillin–streptomycin–glutamine (PSG).

Human mesothelial cells (MeT-5A) were obtained from American Type Culture Collection (ATCC, CRL-9444^TM^, Manassas, VA, USA). They were cultured in Medium 199 (M199, Gibco) supplemented with 10% fetal bovine serum (FBS), 1% penicillin/streptomycin, 3.3 nM epidermal growth factor (EGF) 400 nM hydrocortisone. Human mesothelial primary cells (HMC, #36223-01, Celprogen, Torrance, CA, USA) were grown in RPMI1640 supplemented with 10% fetal bovine serum (FBS), penicillin (100 U/mL) and streptomycin (100 mg/mL). Human Pulmonary Microvascular Endothelial Cells (HPMEC) were purchased from ScienCell Research Laboratories (Carlsbad, CA, USA, 3000-SC) and cultured in complete Endothelial Cell Medium (1001-SC) on bovine plasma fibronectin-coated culture vessel (2 μg/cm^2^, #8248-SC). Immortalized human lung pericytes [55] were cultured in human Pericyte Medium purchased from ScienCell Research Laboratories (Carlsbad, CA, USA, #1201) on a gelatine (0.2%) pre-coated culture vessel. Primary human pulmonary fibroblasts were purchased from PromoCell (St. Louis, MO, USA, #C-12360) and cultured in Fibroblast Growth Medium 2 (#C-23020).

HEK293T cells were cultured at 37 °C under a 5% CO_2_ atmosphere in Dulbecco’s modified Eagle’s medium (DMEM) supplemented with 10% fetal bovine serum (FBS), penicillin (100 U/mL) and streptomycin (100 mg/mL). They were transiently transfected with various plasmid constructs using the calcium phosphate method and collected 48 h after transfection.

### 4.4. Recombinant Protein Expression and Purification

Expi293F cultures (2.5 × 10^6^ cells/mL) were transfected by gently adding a transfection mixture consisting of plasmid DNA (1 µg/mL of culture), and PEI Max 40K diluted in Opti MEM medium (Gibco). After 8–10 h, the cultures were supplemented with 5 mM valproic acid, 6.5 mM sodium propionate, 0.9% (*w*/*v*) glucose, and 50 µM CuSO_4_ and placed in shaking humidified 8% CO_2_ incubator at 30 °C and 80 rpm. The cells were harvested after 72 h by centrifugation and washed with cold PBS. The cell pellet was resuspended in base buffer (50 mM HEPES pH 7.5, 200 mM NaCl, 5% glycerol and 0.5 mM TCEP) supplemented with protease inhibitors (complete Roche, Basel, Switzerland) and disrupted using a cell disruptor (CF 2 model, Constant systems, Daventry, UK). The cell membranes were obtained by ultracentrifugation at 195,000× *g*. Membrane proteins were solubilized in base buffer containing 1% (*w*/*v*) N-dodecyl-β-D-maltopyranoside (DDM, Anatrace, Maumee, OH, USA), 0.1% (*w*/*v*) cholesteryl hemisuccinate (CHS, Anatrace), for 1 h at 4 °C and then ultracentrifuged again. Recombinant Heph was purified from the resulting supernatant with Co^2+^ charged-TALON resin (Takara Bio, San Jose, CA, USA). After extensive washing, the protein was eluted with base buffer containing 300 mM imidazole and 0.03% DDM/0.003% CHS. Imidazole was removed using a desalting column (CentriPure100-Z25M, Emp Biotech, Berlin, Germany), and the protein was incubated overnight with HRV 3C protease to remove the tag. After incubation with TALON resin, flowthrough containing the cleaved protein was collected, concentrated, and further purified by size exclusion chromatography (Superdex G200 increase column, Cytiva, Marlborough, MA, USA). Protein purity was assessed by SDS-PAGE and concentration was determined by absorbance at 280 nm (ε280 200,000 M^−1^ cm^−1^).

### 4.5. Ferroxidase Activity Assay

Heph ferroxidase activity assays were performed at 25 °C in 50 mM HEPES pH 7.5, 200 mM NaCl, 0.02% DDM and 0.002% CHS. Upon addition of 60–300 µM FeSO_4_ to 0.3 µM Heph, Fe^2+^ oxidation was measured at 315 nm using a UV-Vis microplate spectrophotometer (CLARIOstar plus, BMG labtech, Ortenberg, Germany). Corrections for iron auto-oxidation were performed by subtraction of traces obtained in the absence of enzyme. All measurements were carried out in triplicate to generate the error bars. Analysis of the time course data to extract kinetic parameters was performed with iFIT (https://www.i-fit.si/) [55].

### 4.6. Immunocytochemistry and Proximity Ligation Assay (PLA)

Human lung pericytes and HEK293T cells transfected with the indicated plasmid DNAs (24 h after transfection) were fixed with 3% PFA for 20 min at room temperature (RT). Permeabilization, quenching and blocking were performed by incubating the cells in 1% BSA, 0.1% Triton X-100 and 50 mM glycine in PBS, for 30 min at RT. Antibody staining was performed by standard procedures. GFP was visualized by autofluorescence. Nuclei were stained with DAPI (Sigma-Aldrich, 1:1000) for 5 min. Coverslips were mounted with Fluorescence Mounting Medium (Dako, Glostrup, Denmark).

PLA cells were seeded onto glass coverslips and transfected with the indicated plasmid DNAs. One day after transfection, the media were replaced with DMEM containing no FBS in order to remove exogenous Tfn. The next day, the cells were incubated with 0.25 µM apo-Tfn and holo-Tfn solutions for 30 min and then washed to proceed with PLA following the manufacturer’s instructions (Sigma-Aldrich Duolink, #DUO92102). All images were acquired using a Leica DM3000 microscope (Leica, Wetzlar, Germany) and a Leica DFC320 digital camera.

### 4.7. Surface Biotinylation, Immunoprecipitation and Western Blot Analysis

The biotinylation assay was exploited to examine the expression and surface targeting of endogenous Heph in all human primary cell lines tested. Briefly, cells grown on 10 cm dishes were incubated with 0.5 mg/mL EZ-Link Sulfo-NHS-LC-Biotin (Pierce, Appleton, WI, USA) in PBS at 4 °C for 30 min. To quench the reaction, the cells were washed three times with cold PBS containing 0.1 M Tris-HCl pH 7.4. The cells were harvested by centrifugation at 1000× *g* for 10 min and then lysed in RIPA buffer (25 mM Tris-HCl, pH 7.6, 150 mM NaCl, 1% NP40, 0.5% sodium deoxycholate, 0.1% SDS, 10% glycerol) supplemented with protease inhibitor cocktail (Roche). The collected lysates were then incubated with Neutravidin Agarose Resin (Pierce, #29200) for 2 h at 4 °C. The beads were then washed three times with lysis buffer and eluted with SDS loading buffer.

In co-immunoprecipitation (Co-IP) experiments, transfected HEK293T cells grown in 10 cm Petri dishes were treated with two different crosslinkers before being harvested as previously described. The cross-linker Lomant’s Reagent (DSP, Pierce) is a cell-permeable chemical crosslinker that reacts with amino groups such as those of lysine residues, and it is able to crosslink Heph/Fpn1 complexes localized at different membrane compartments. The membrane-impermeable Dithiobis-(sulfosuccinimidylpropionate) (DTSSP, Pierce) was used to evaluate the fraction of Heph/Fpn1 interaction selectively occurring at the plasma membrane. DSP was dissolved in DMSO at 10 mM, then mixed in PBS Plus to a final concentration of 0.5 mM; DTSSP was directly dissolved in the appropriate volume of PBS at the final concentration of 1.5 mM. The DSP treatment was performed by incubating transfected cells for 30 min at 4 °C; DTSSP treatment was performed upon 2 h incubation at 4 °C. Quenching was carried out by washing the cells with 200 mM Tris/HCl, pH 7.6 solution 3 times, 5 min each. The cells were then harvested and lysed in RIPA buffer as previously described. Then, 1/10 of the whole cell lysate volume was mixed with 2X Laemmli sample buffer and used as the reference input. The GFP-trap or DYKDDDDK Fab-Trap resin (Chromotech, Martinsried, Germany, #gta and #ffa, respectively) were equilibrated in RIPA buffer and incubated with cell lysates in rotation end-over-end for 1 h at 4 °C. The beads were then washed 3 times in lysis buffer and the proteins were eluted by adding 2X Laemmli buffer with DTT. Samples derived from surface biotinylation and co-IP were fractioned by 8% SDS-PAGE under reducing conditions and transferred to a nitrocellulose membrane. Primary antibodies diluted in 5% skimmed milk in TBST (10 mM Tris, pH 8.0, 150 mM NaCl, 0.5% Tween 20) were incubated over-night and revealed by HRP-conjugated secondary antibodies (Sigma) followed by ECL (Advansta, San Jose, CA, USA).

### 4.8. Gene Expression Analysis

RNA was extracted using the PureLink RNA Mini Kit (Invitrogen, #12183018A) according to the supplier’s instructions. The RNA concentration and purity were determined spectrophotometrically with a 260/280 ratio above 1.8. Then, 1 µg of RNA was reverse-transcripted to cDNA using SuperMix kit (Bioline, London, UK) and qPCR was carried out on a Rotor-Gene 6000 (Corbett, Qiagen, Milan, Italy) using SYBR™ Green PCR Master Mix (Applied Biosystems, Milan, Italy). Each sample was analyzed in triplicate, and non-reverse-transcribed RNA and water served as negative controls. The PCR conditions were 95 °C for 10 min, followed by 40 cycles of 95 °C for 30 s, 60 °C for 30 s, and 72 °C for 30 s. All primers (Table S1) were designed using Primer3Plus software (version 3.3.0) according to NCBI, Ensembl, and FANTOM-CAT sequence databases. For relative quantification, the GAPDH was used as the internal standard reference. All experiments were performed at least in duplicate technical replicates. The analyses were carried out using the ΔCT (threshold cycle) method.

### 4.9. Statistics

Statistical analyses co-IP and qRT-PCR were conducted using GraphPad Prism software (Version 10.0.0). Differences between diverse groups were evaluated using one-way or two-way analysis of variance (ANOVA) followed by appropriate post hoc tests for multiple comparisons. A *p*-value of less than 0.05 was considered statistically significant. Data were expressed as ±Standard Error of the mean (SEM). All experiments were performed in at least triplicate to ensure reproducibility.

## 5. Conclusions

In the present study, we provide a mechanistic understanding of how changes in iron handling due to defects in sensing local iron requirements may influence individual responses to environmental carcinogens. Our results contribute to a better understanding of the genetic contributions to asbestos-related cancers and highlight the potential for identifying protective biomarkers that could serve as the basis for targeted prevention and intervention strategies.

## Figures and Tables

**Table 1:** Kinetic parameters for Heph ferroxidase activity.

Hephaestin	K_m_	SEM	V_max_ (µM/min)	SEM
WT	7.71	1.55	10.60	0.80
D568H	5.38	0.97	10.30	0.60

## Data Availability

The data presented in this study are available in this article and the Supplementary Materials. Further inquiries can be directed to the corresponding authors.

## Supplementary Materials

The supporting information can be downloaded at: https://www.mdpi.com/article/10.3390/ijms26062607/s1.

## Abbreviations

The following abbreviations are used in this manuscript:

Apo-Tfn	Transferrin (protein), Iron-Depleted Form
BBB	Blood–Brain Barrier
CP	Ceruloplasmin
CT	Threshold Cycle
FBS	Fetal Bovine Serum
Fpn1	Ferroportin
FTH1	Ferritin Heavy Chain
GFP	Green Fluorescent Protein
GPI	Glycosylphosphatidylinositol
HEK293T	Human Embryonic Kidney Cell
HEPH	Hephaestin (gene)
Heph	Hephaestin (protein)
HMC	Human Mesothelial primary cell
Holo-Tfn	Transferrin (protein), iron-loaded form
HPMEC	Human Pulmonary Microvascular Endothelial Cells
Huvec	Human Umbilical Vein Cell
LC	Lung Cancer
MeT-5A	Human Mesothelial Cells
MPM	Malignant Pleural Mesothelioma
PCR	Polymerase Chain Reaction
PLA	Proximity Ligation Assay
qRT-PCR	Quantitative Real-Time PCR
ROS	Reactive Oxygen Species
SNP	Single Nucleotide Polymorphism
TF	Transferrin (gene)
TfnR	Transferrin Receptor
TT1	Immortal Alveolar Type-1-Like Cells
