# Supplementary material for: Functional Characterization of the Hephaestin Variant D568H Provides Novel Mechanistic Insights on Iron-Dependent Asbestos-Induced Carcinogenesis
Source: Int J Mol Sci. 2025 Mar 13;26(6):2607. doi: 10.3390/ijms26062607 (PMC11941830; doi:10.3390/ijms26062607)
Supplement: Supplementary file 1 [file ijms-26-02607-s001.zip › ijms-3487993-supplementary.pdf]

SUPPLEMENTARY DATA:

Supplemental Figure S1: HephD568H is delivered at the plasma membrane just like HephWT

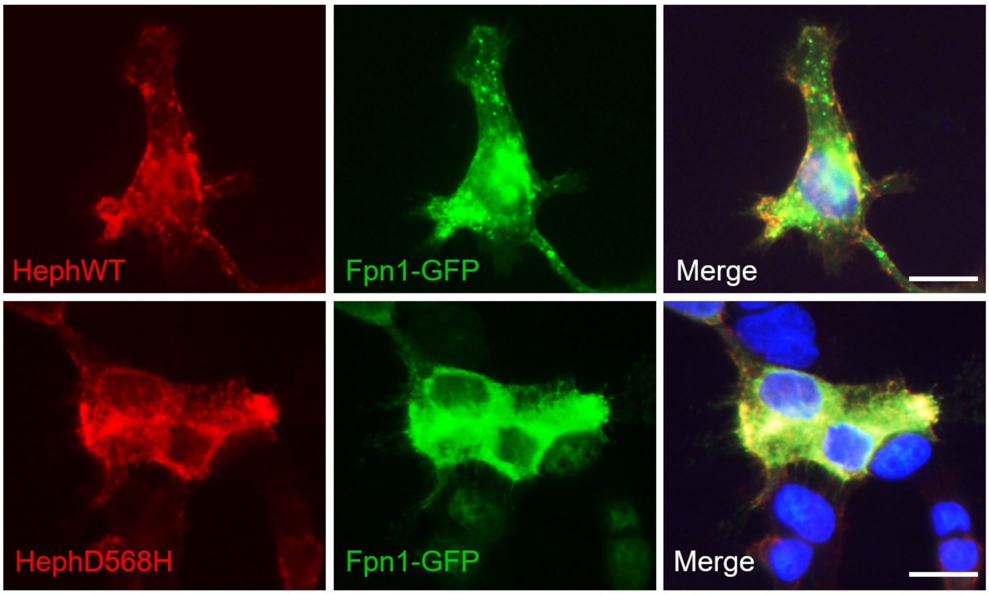

Representative images of HEK293T cells co-transfected with HephWT or D568H together with Fpn1-GFP. Surface staining was performed by incubating the cells with the primary anti-Heph antibody diluted in ice-cold medium for 1 h at 4°C. Cells were then fixed and incubated with anti-mouse Alexa 594 secondary antibody. Under these conditions, antibody induced Heph internalization is strongly hampered thus allowing a selective surface localization detection. Surface immuno-labeling attests the presence of HephWT and HephD568H at the plasma membrane, as expected for a ferroxidase, and partially co-localizing with the permease Fpn1. Nucleus was stained with 4, 6-diamidino-2-phenyl indole (DAPI) in blue. Scale bar: 10 µm

Supplemental Figure S2: Proximity Ligation assay controls

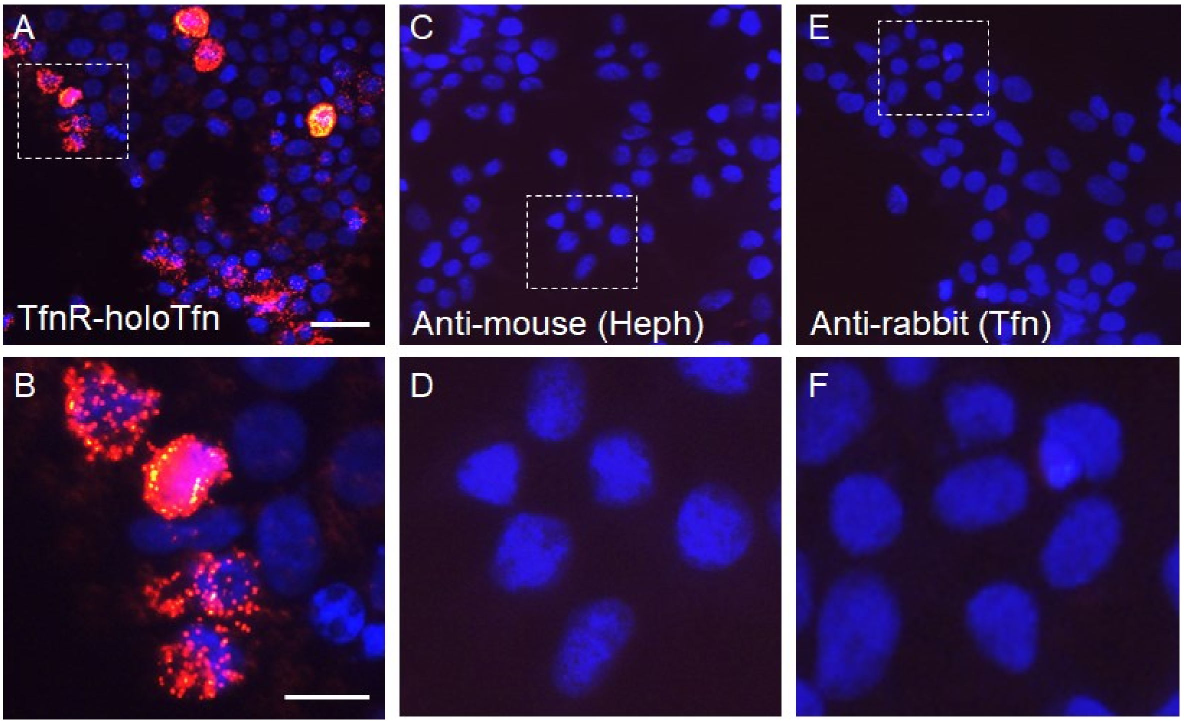

Representative images of PLA controls. The positive control consisted of probing a known interaction as the one occurring between transferrin receptor (TfnR) and holo-Tfn (panel A). Then, each antibody used to probe for Heph (anti-mouse, panel C) and Tfn (anti-rabbit, panel E) was assessed alone for non-specific binding thus ensuring that the antibodies used did not produce PLA signal. Panels B, D and F represent higher magnifications of the corresponding dashed area indicated on the corresponding upper panel. Scale bar 20 µm panels A-C; scale bar 10 µm panels B-F.

Supplemental Figure S3 Heph expression over cell lines representative of the lung and pleural district.

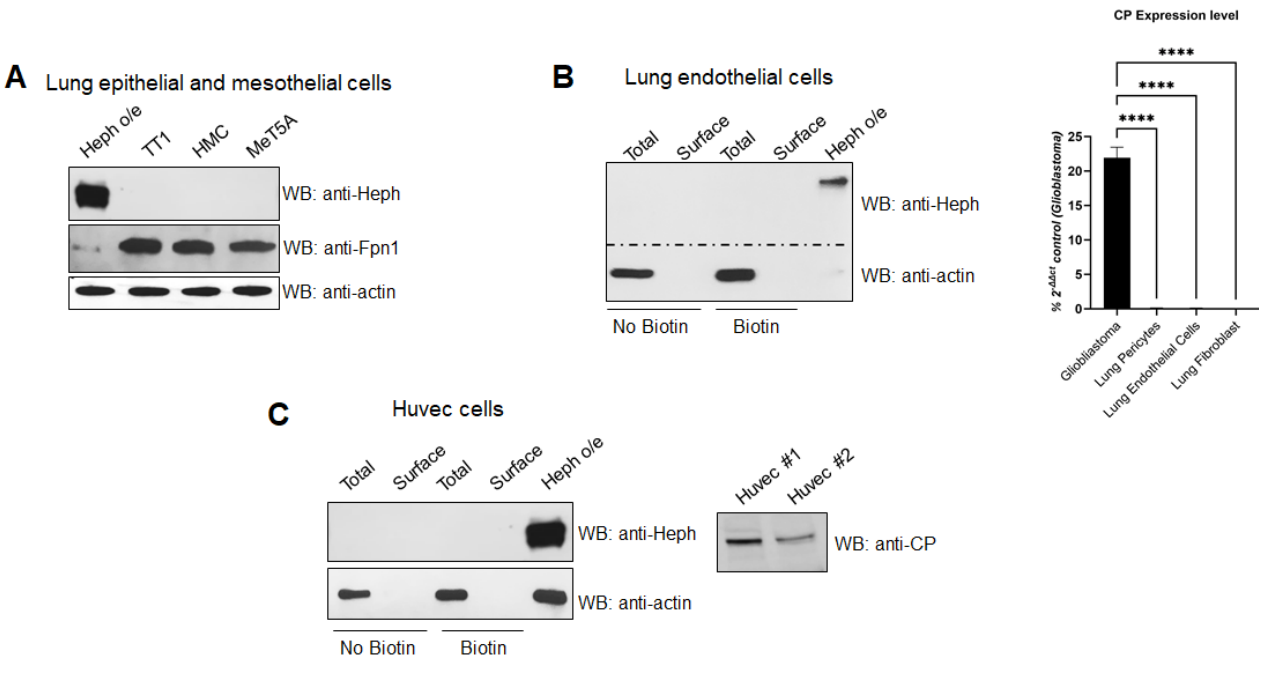

A) Human immortal alveolar type 1(AT1)-like cell line (TT1), human mesothelial primary cell line (HMC) and MeT5A were lysed in RIPA buffer and 20 µg total protein was run on an 8% SDS-PAGE. Nitrocellulose membrane was probed with anti-Heph monoclonal antibody, anti-Fpn1 (31A5 monoclonal) and anti-actin HRP-conjugated. While all lines tested express the permease Fpn1, none of them expresses Heph. B) Lung microvascular endothelial cells were subjected to surface biotinylation and probed for Heph expression (membrane targeting). Western blot analysis indicate that these cells lack Heph expression while qRT-PCR attests that they do not express CP (n=4, data are expressed as mean ± SEM, p-value ****<0,0001). RNA extracted from the glioblastoma cell line (Merck, #U-373 MG) was included as positive control. C) Umbilical vein endothelial cells (Lonza) were also tested as described in B. These cells rely on CP for ferroxidase activity.

Supplemental Table S1

Gene	Tm	sense	sequence	Accession Number
**GAPDH**	60ºC	Forward<br>Reverse	5'-CTCTGCTCCTCCTGTTC-3'<br>5'-GCCCAATACGACCAAATCC-3'	NM_002046.7
**HEPH (Hephaestin)**	60°C	Forward<br>Reverse	5'-GCTATGCTCTTTCCTCATACC-3'<br>5'-CCTGCTTCTCGATGGCT-3'	NM_001367233.
**TF (Transferrin)**	60°C	Forward<br>Reverse	5'-GAGAAAATGCTCCACCTCAT-3'<br>5'-ACTCATGGATCATCTGCGTTC-3'	NM_001063.4
**CP (Ceruloplasmin)**	60.°C	Forward<br>Reverse	5'-CTTCCAATACAAGCACAGGG-3'<br>5'-CATGGCAGTGGAGTAACCA -3'	NM_000096.4
**FTH1 (Ferritin Heavy Chain)**	60°C	Forward<br>Reverse	5'-GTTTACCTGTCCATGTCTTACT-3'<br>5'-TCTCAGCATGTTCCCTCT-3'	NM_002032.3
